# Allergic Diseases: A Comprehensive Review on Risk Factors, Immunological Mechanisms, Link with COVID-19, Potential Treatments, and Role of Allergen Bioinformatics

**DOI:** 10.3390/ijerph182212105

**Published:** 2021-11-18

**Authors:** Fahad M. Aldakheel

**Affiliations:** 1 Department of Clinical Laboratory Sciences, College of Applied Medical Sciences, King Saud University, Riyadh 11433, Saudi Arabia; faldakheel@ksu.edu.sa; Tel.: +966-56-300-0633; 2 Prince Sattam Chair for Epidemiology and Public Health Research, College of Medicine, King Saud University, Riyadh 11461, Saudi Arabia

**Keywords:** allergy, immune system, microbiota, allergology, allergen bioinformatics

## Abstract

The prevalence of allergic diseases is regarded as one of the key challenges in health worldwide. Although the precise mechanisms underlying this rapid increase in prevalence are unknown, emerging evidence suggests that genetic and environmental factors play a significant role. The immune system, microbiota, viruses, and bacteria have all been linked to the onset of allergy disorders in recent years. Avoiding allergen exposure is the best treatment option; however, steroids, antihistamines, and other symptom-relieving drugs are also used. Allergen bioinformatics encompasses both computational tools/methods and allergen-related data resources for managing, archiving, and analyzing allergological data. This study highlights allergy-promoting mechanisms, algorithms, and concepts in allergen bioinformatics, as well as major areas for future research in the field of allergology.

## 1. Introduction

Allergies are chronic, inflammatory disorders with aberrant immune reactions to certain environmental chemicals, which are called allergens. A number of proteins from distinct origins can behave as allergens responsible for allergic reactions from different environments [1]. Allergy symptoms range from miserable to life-threatening reaction danger. The allergic reaction develops when the immune system is exposed to a relatively harmful antigen, according to renowned allergy experts [2]. Allergies include a wide variety of reactions. Atopy, on the other hand, is a genetic predisposition to diseases in which immunoglobulin (IgE) antibodies are produced in response to even minor exposure to environmental triggers that do not bother most people. Therefore, every atopic reaction is an allergy [3]. A variety of chemical allergens (e.g., dyes, creams, fragrances in the hair, and skincare products), food allergens (e.g., genetically modified foods, tree nuts, peanuts, and eggs), and aeroallergens (e.g., dust mites, spores, pollens) can cause allergic symptoms such as skin reactions, anaphylaxis allergic rhinitis, and asthma [4,5,6].

Clemens von Pirquet, a Viennese doctor, coined the term “allergy” in 1906 after observing the hypersensitivity of his patients to typically harmless substances, such as specific foods, pollen, or dust [7,8]. Previously, allergies were used to describe a wide range of inappropriate inflammatory hyper-immune sensitive reactions. An excessive activation of specific immune system cells that induce inflammation was thought to be the root of the majority of cases. An allergic IgE mediated mechanism was later discovered to disproportionately activate specific immune system cells and to release inflammatory mediators [8]. Philip Gell and Robin Coombs proposed a new categorization system in 1963 that included immunological components and the immune process in order to identify reactions of type I to IV hypersensitivity [9,10]. Acute IgE-mediated type I hypersensitivity was referred to as “allergy” in this classification system. This features the rapid onset of hypersensitivity or allergy symptoms and develops reactions within less than 20 min after allergic exposure. Isolation and a description of the significance of IgE were the key findings of the allergy mechanism [11,12]. Kimishige Ishizaka and his colleagues originally found out in 1960 that the antibody class IgE mediates type I allergic hypersensitivity. The IgE, also known as regenic antibody or allergic antibody, was the key immunological component that might cause atopy or allergy in immune-compromised people [12].

This article will provide an overview of the risk factors and treatment for allergy disorders, as well as the allergy-promoting mechanism of viruses and bacteria and the role of allergen bioinformatics approaches in overcoming this problem.

## 2. Signs and Symptoms

**Figure 1:**
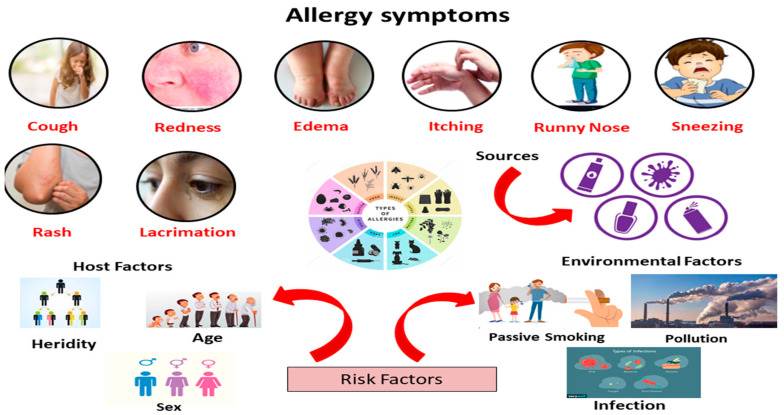
Symptoms and risk factors of allergic diseases.

Allergens are protein molecules found in various forms in a variety of substances. Multiple organ systems are affected by allergens, including the circulatory, cardiac, digestive, and respiratory systems. Allergens can produce edema, cutaneous reactions, hypotension, bronchoconstriction, death, and coma depending on the sensitization rate and severity. The sudden, life-threatening, and extreme hyper-immune response is known as anaphylaxis and can cause death if not treated. Numerous allergenic compounds, such as latex, can cause skin rashes and irritations, resulting in angioedema contact and dermatitis. Allergens vary in nature and source, causing moderate to severe systemic and cutaneous symptoms depending on the exposure mechanism and route of sensitization. These can be inhaled, ingested, or exposed through skin contact. Many pollen and dust allergens are microscopic airborne particles [13]. These are easily inhaled and cause symptoms in organs exposed to the allergen, such as the nose, lungs, and eyes. Mucosal irritation, a runny nose, and sneezing are the most common symptoms of allergic rhinitis (hay fever). Swelling, irritation, and redness in the eyes are all possible side effects. Allergy particles inhaled into the lungs can cause bronchial hyper responsiveness. Particular airborne allergens can be inhaled in the lungs and induce asthmatic symptoms. Coughing, bronchoconstriction, and sneezing are caused by the narrowing of the airways. The increased mucus production restricts airflow to the lungs and thickens the airways, causing a shortness of breath (bronchial hyperresponsiveness, wheezing, and dyspnea). Allergic reactions can also be triggered by the ingestion of medications and food, allergen contact, drug administration, and insect bites [14]. Food and contact allergies symptoms include hives, itchy and swollen skin, edema, vomiting, gastrointestinal discomfort, and diarrhea. Food allergies rarely result in rhinitis or respiratory (asthmatic) reactions [15]. Insect bites, drugs, medicines, and insect contact with venom lead to systemic allergic responses affecting several organs (Figure 1) [16].

## 3. Epidemiology

Globally, the prevalence of allergic diseases is rising rapidly in both developing and developed countries. Some studies demonstrate that, in developed countries, allergy disorders are significantly more prevalent compared to developing countries [17,18]. The prevalence of allergies or allergic diseases is determined by several factors that determine the population’s susceptibility to developing atopic conditions. The basis for allergy incidence in individuals is a mainly genetic and environmental predisposition. A total of 8–10% of the global population suffer from one or more allergic diseases, ranging from mild rhinitis to severe anaphylaxis or asthma [18]. The prevalence and causes of these allergies are summarized in Table 1.

## 4. Causes and Risk Factors of Allergy

Allergens, which can be found in a variety of environments, were found to be the causative agents for allergy or hypersensitivity reactions. Recognizing allergy risk factors is critical for identifying modifiable factors and individuals who may benefit from preventive measures. Risk factors can be primary, affecting the atopic disease incidence, or secondary, affecting allergic sensitization or triggering symptoms in someone already sensitized. Allergy risk factors are divided into two categories: host and environmental (Figure 1) [26].

### 4.1. Host Factors

Race, sex, heredity, and age are the host characteristics that influence the allergy risk, with heredity being the most important. Host factors are not currently modifiable.

#### 4.1.1. Race

Racial disparities in the occurrence of hay fever and asthma are difficult to explain because it is difficult to separate environmental impacts and changes produced by migration from racial factors. Black people have higher levels of IgE than Caucasians [27]. There have been reports of racial differences in the outcomes of allergic diseases, with African Americans suffering disproportionately more than white people [28,29]. Fish and shellfish allergies and a higher risk of wheat allergy are significantly more prevalent in black children than white children. The risk of deadly anaphylaxis for black children is two to three times more than that of white children [30].

#### 4.1.2. Heredity

An allergic condition can be inherited; the development of allergic diseases has a strong genetic basis. A total of 70% of homozygous twins and 40% of non-identical twins reported similar allergy problems. Allergic people have been observed to have children with comparable allergic conditions and serious symptoms [31]. Immune sensitivity is more prevalent in allergic parents than non-allergic parents. The most prevalent allergy diseases have been discovered to be hereditary. The likelihood of developing allergies seems to be genetic and associated with a malfunction in the immune system. A total of 60–80% of biparental allergic children, 30–50% of single parental allergic children, and 12% of children with no allergic family history will develop allergic disease [32,33].

#### 4.1.3. Sex

Atopy is predominant among boys rather than girls. This sex difference can be explained by a higher sensitization rate in men compared to women for cat epithelium, grass pollen, and house dust mite. This may also explain why boys have a higher chance of developing asthma. Although this gender disparity diminishes with age, most authors report that men have a higher prevalence of specific IgE antibodies, skin test positivity, and higher total IgE levels than women. However, for several atopic disorders, at least for asthma, the prevalence of disease appears to reverse in young adulthood [34,35].

#### 4.1.4. Age

Age affects the likelihood of allergic sensitization and atopic disease. Allergic sensitivity is high in children, especially children with an atopic history. IgE levels in infancy are at maximum and reduce quickly between 10 and 30 years of age; following that, the decrease slows down progressively [36]. Asthma is more prevalent in children below ten [37], and hay fever is most common in young adults and children [38]. Eczema is a childhood disease that begins before the age of five in 87% of adult eczema patients and has frequent remission before adulthood [39]. The prevalence of gastrointestinal allergy disease is higher in toddlers and infants [40].

### 4.2. Environmental Factors

Some environmental allergens are modifiable and have been the target of preventive measures. Immune modulation occurs as a result of environmental changes, which favors allergy disease development in susceptible populations. Significant environmental factors influence immune sensitization, resulting in atopy [41].

#### 4.2.1. Passive Smoking

There is evidence that passive smoking raises serum total IgE levels and increases the risk of allergic diseases, such as allergic rhinitis, asthma, and atopic dermatitis. Passive smoking is undeniably a significant asthma risk factor [41]. The associated allergy conditions with smoking exposure have been evaluated in several studies. The results were contradictory and alternated among the protection [42,43] and negative effects of smoking [44,45] in every allergic condition; however, some studies did not discover any effects [46,47].

#### 4.2.2. Pollution

Human, animal, and epidemiological studies all indicate that air pollutants play a significant role in the aetiology of allergic diseases, such as asthma, in terms of exacerbation and development. This involves gaseous elements, such as particulate matter (PM), ozone (O3), and nitrogen dioxide (NO2), produced by industry and automobiles [48]. NO2 can significantly raise the allergic response to inhaled allergens, according to asthmatic controlled-exposure studies [49]. O3 exposure has also been linked to an increase in worsening symptoms, respiratory infections, hospital admissions, and the need for rescue medication, peak flow rate reductions, and asthma attacks [50,51,52,53]. Gauderman et al. observed a greater chance of developing asthma for children in high O3 areas [54], while Ackermann-Liebrich et al. documented the lifelong history of physician-diagnosed asthma as a result of outdoor residential NO2 levels [55]. Many researchers have looked into the relationship between airborne traffic-related pollutants and asthma in metropolitan regions. Asthmatic children in Mexico City are highly correlated with respiratory symptoms and traffic-related air pollution [53]. Three birth cohorts’ studies were carried out by children in Germany, the Netherlands, and Sweden until the age of four or six and suggest a favorable link between medically diagnosed asthma and traffic pollution [3,56,57].

#### 4.2.3. Dietary Habits

It is well known that, in addition to exposure to allergens and poor hygiene habits, environmental pollution and tobacco smoke, being overweight, having a low-quality diet, obesity, and a high caloric intake in adolescents and children are important environmental factors that are responsible for developing allergies [58,59]. The majority of kids with respiratory allergies, particularly asthmatics, reported poor eating behaviors, such as eating and snacking before sleep. A reduced intake of vegetables and fruit with anti-inflammatory and antioxidant properties is likely to adversely affect the prevalence/management of asthma [60]. Results from cross-sectional [61,62,63] and case-control [60,64] studies indicate that fast food consumption is significantly associated with allergic rhinitis (pollen fever) and asthma. According to Wang et al., the proportion of processed foods consumed is related to the severity and frequency of asthma attacks [65]. Diets high in vegetables, cereals, and starch have been linked to a lower risk of allergic rhino conjunctivitis [66] and asthma [67]. Evidence reveals that high-fat diets and foods are frequently associated with rhinitis [68,69], asthma [63,67,70], respiratory health [71], and allergies, though some studies have not found these associations [72].

#### 4.2.4. Infections

Bronchial asthma and allergic sensitization are linked to respiratory infections, particularly viral infections. Childhood allergic sensitization, followed by wheezing respiratory tract illnesses caused by respiratory pathogen infections, appears to be reproducible and consistent among the various environmental risk factors implicated in the development of childhood asthma [73,74,75]. Preschool wheezing illnesses caused by both viral [76,77,78] and bacterial [79,80,81] pathogens are also linked to an increased asthma risk and recurrent wheezing. The role of bacterial and viral infections in the development of allergic diseases is discussed later.

## 5. Mechanism of Allergy and Immune System

The role of the immune system is to protect the body against invading pathogens causing different diseases. When the immune system misidentifies a harmless foreign antigen as a pathogen, an allergic reaction occurs [82]. To protect the organism against exaggerated stimulation signals from harmless antigens, such as environmental and self-antigens, the immune system must be closely monitored. In genetically predisposed individuals, an imbalance in the immune system’s regulatory mechanisms may lead to allergic diseases or autoimmune disorders, depending on the nature of the antigen [83,84].

**Figure 2:**
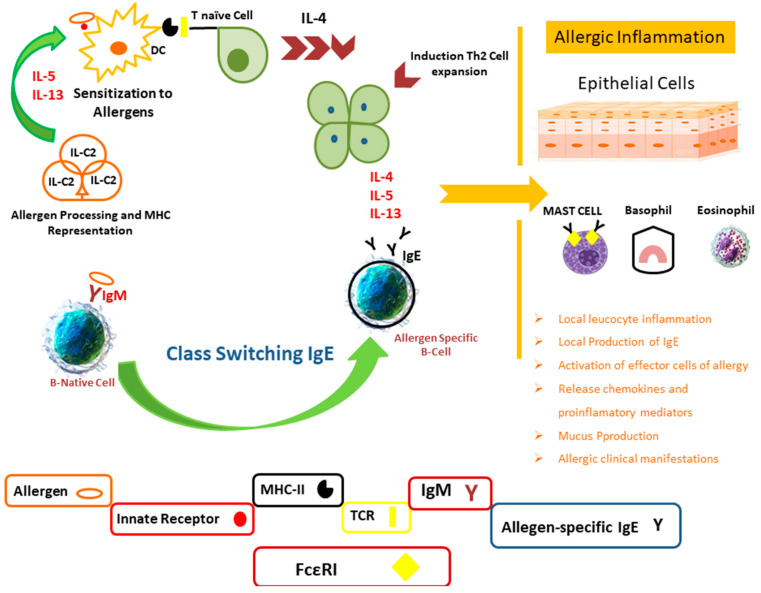
Allergic reaction mechanisms.

During an allergic reaction, the immune system must detect pathogenic stimuli and generate a robust immune response. Specific antigen sensitization is required: naive T and B cells identify specific sections of antigens, which are termed epitopes. First, specific MHC (major histocompatibility complex) class II antigens synthesized on the antigen-presenting cells (APC) surface detect allergens and deliver them to naive T lymphocytes. T cell activation causes T helper type 2 (T_H_2) cells to proliferate and differentiate. Interleukin IL-5, IL-4, and IL-13 and innate (ILC-2) lymphoid cells that can maintain and enhance local T_H_2 inflammation caused by the secretion of T_H_2 cytokines (IL-13 and IL-5) are the primary cytokines responsible for the allergic response [85]. These ILs act on B cells, causing them to switch to the Ig class E (IgE). Allergen-specific IgE antibodies bind to high-affinity IgE receptors (FcRI) on basophils and mast cells. Repeated exposure to the allergen causes FcRI-bound IgE to crosslink, boosting the release of other mediators and histamine that generate allergic disease symptoms. Allergen-specific cells are enlarged and reactivated locally after 6–12 h of allergen exposure, culminating in the late phase of an allergic reaction. Effector cells (basophils, mast cells, and eosinophils in particular) release cytokines and inflammatory mediators, prolonging the proinflammatory response (Figure 2). The symptoms of allergic disorders are caused by this phase, and persistent allergen exposure causes the disease to become chronic [83,86].

Specific antigen sensitization is required for allergic diseases development. Inflammatory cytokines (IL-13, IL-4, and IL-5) are produced as a result of cell expansion and differentiation to T_H_2 cell subtypes. They regulate the activation and recruitment of pro-inflammatory cells (mast cells and eosinophils) in mucosal target organs, as well as the class switching of IgE in B cells. Allergy symptoms and inflammation are triggered by these activations [87].

## 6. Allergy and Microbiota

The microbiota (intestinal microflora) are a collection of microorganisms, primarily bacteria, that form a complex ecosystem in the human digestive tract. Microbiota are influenced by a wide range of environmental and nutritional factors, and play a complex role in allergic diseases. According to a recent study, gut microbiota has a substantial impact on immune system development. The gut microbiota plays a significant role in the formation of immune system organs and help to identify host immune response patterns. According to research on the relationship between gut microbiota and immune diseases, modifications in commensal bacteria can trigger immune system changes that affect immune system maturation, oral tolerance development, and host metabolism regulation [88,89]. Due to the fact that the immune system is regulated by the normal intestinal ecosystem, the risk of allergy or atopy is likely to increase as the dysbiosis of the gastrointestinal tract worsens. Dysbiosis is described as a disruption in gut homeostasis caused by a change in the function and composition of the microbiota [90]. Numerous studies suggest that dysbiosis intestinal, or quantitative and qualitative abnormalities in the microflora composition, may be a factor in the pathogenesis of a variety of disorders, including inflammatory bowel disease, necrotizing enterocolitis in newborns (NEC), celiac disease, irritable bowel syndrome, atopic dermatitis, allergic disorders, cancer, depression, and others [91,92,93]. Many atopy and allergy patients have altered microbiota [94], as evidenced primarily by stool microbiota analysis [95,96,97]. Dysbiosis has also been found in the lower and upper respiratory tract microbiota of asthma patients [98], as well as the skin microbiota of atopic dermatitis patients [99,100], and in the gastrointestinal tract of food allergy sufferers [101,102].

There is mounting evidence that dysbiosis precedes the onset of allergic symptoms. A lack of specific bacterial species from the gut microbiota among infants aged 1–3 months was linked to a higher risk of developing a recurrent wheeze, asthma, or atopy later in life, according to birth cohort studies [103,104]. Such changes were associated with reduced levels or a lack of anti-inflammatory polyunsaturated fatty acids [104]. Proteobacteria, especially Haemophilus spp., are more prevalent in asthmatic adults’ lungs than in healthy controls, who have a higher proportion of Bacteroidetes. Furthermore, asthmatic children have a higher abundance of Proteobacteria than healthy controls [105]. Intestinal dysbiosis in egg-allergic children was marked by an increase in the Lachnospiraceae and Streptococcaceae genera, as well as a decrease in the Leuconostocaceae families, when compared to non-food-allergic controls [106]. Ege et al. compared the microbial data of 489 school-aged children from rural and urban areas in Germany and found a number of bacteria, including Lactobacillus, Staphylococcus, and Acinetobacter, that were inversely related to asthma and hay fever [107]. When children with allergic airway diseases were compared to children from similar surrounding environments, such as both from urban areas, a mild reduction in microbiota diversity was observed, and microorganisms from the phylum Firmicutes were significantly less expressed than in healthy children [108]. A similar pattern was observed among Swedish children. At infancy, children with asthma have a lower diversity of gut microbes than children without asthma [109].

Breast milk provides immune factors, such as IgA antibodies, that protect against a variety of health problems in infancy, including obesity and being overweight, necrotizing enterocolitis, diabetes, infections, and allergic disease [110,111], as well as reducing the risk of diseases later in life [112]. Breastfeeding, on the other hand, has been the subject of debate in the literature regarding its ability to protect children from developing asthma and allergic disease [113]. Epidemiological studies in the debate over whether breastfeeding can protect against allergic disease and asthma in early childhood provide contradictory results [112]. While breastfeeding is advised for all infants, regardless of allergic history [114], with protective effects of breastfeeding on asthma reported in young children [115,116], other studies of children at low [117] or high risk [118,119], or adults [120], have found no protective effects.

Dysbiosis may start even earlier, according to recent research, as meconium from at-risk neonates displays an altered microbiota-derived metabolome and delayed gut microbial diversity, characterized principally by a lack of anti-inflammatory fecal lipids [121]. This dysbiosis is significantly linked to parental sickness, implying that maternal health during pregnancy may have an impact on the vertical transmission of microbes that affect early microbiota development. Changes in the intestinal microbiota composition may induce food allergy resistance or vulnerability via a microbially responsive FOXP3+ RORt + T_reg_ cell subset, which is known to be critical for food tolerance maintenance [122].

Mouse studies, particularly experiments with germ-free mice, provide experimental evidence for the association of microbiomes with allergy development. Germ-free mice have an adaptive immune response profile reprogrammed. They are especially predisposed to T_H_2 cell development [123,124]. Germ-free mice may be reconstructed by specific microbial strains and allergy protection is induced by Clostridia and other allergy-protective-related species through IgA production, T_reg_ cell induction, and other immunologic effects [125,126].

It was recently demonstrated that germ-free mice colonized with healthy infants commensals, but not colonized with commensals from cow’s milk allergic infants, were protected against anaphylactic responses to cow’s milk allergens. The Clostridia member, *Anaerostipes caccae*, was further found in this model as protection against allergic response to food [127]. In another food allergy model, colonization with seven species of *Bacteroidales consortium* or a *Clostridiales consortium* suppressed food allergy in the mouse model [128]. Further studies revealed that commensals activated the MyD88–RORt pathway, resulting in the development of T_reg_ cells [128]. These significant experiments aid in a better understanding of the functional significance of dysbiosis in patients with food allergies [129].

Hence, we can conclude that environmental and dietary changes cause dysbiosis in the gut, skin, and/or lung microbiome, resulting in quantitative and qualitative alterations in the microbiota that directly alter immunological pathways implicated in allergic disorders prevention. More research is needed, however, to determine the cause-and-effect link between the microbiota and asthma/allergy clinical phenotypes.

## 7. Viral Infections in Allergy

Viral infections can have a variety of opposing effects on allergy and asthma development; depending on the circumstances, viruses can either protect against or trigger allergic disorders. During the first year of life, the immune system and respiratory tract mature quickly, and postnatal lung development is influenced by and affects viral infection responses. The type of virus, age, intensity, timing, and location of the infection, as well as interactions with pollutants or allergens, have all been linked to allergic diseases development, particularly asthma, regarding viral infections [130]. By binding to certain receptors on the airway epithelial cells surface, viruses trigger antiviral and inflammatory responses, resulting in the innate immune responses activation, the recruitment of mononuclear and neutrophil cells to the area, and the release of mediators, such as chemokines and cytokines [130,131]. Such events can alter immunological and epithelial responses to a hyperactive state [132].

Viral respiratory tract infections and allergens can interact in a variety of ways, including through a flawed epithelial barrier function. Viral respiratory tract infections are related to an impaired innate immunity, suppressed antioxidant properties, and disrupted tight junctions, which may result in a hypersensitivity to allergens and infections [133]. Asthma development has also been linked to viral infections. Asthma onset in childhood and asthma exacerbations in adults and older children are linked to viral respiratory tract infections. The respiratory viruses linked to asthma include those that cause influenza-like illnesses, the common cold, and bronchiolitis, as well as wheeze in children. In school-aged children and adults, respiratory viruses represent approximately 85% of exacerbations of asthma [134,135]. The respiratory syncytial virus (RSV), human rhinoviruses, and influenza viruses are among the viruses linked to asthma exacerbation. Not only can RSV infections induce asthma but, according to an epidemiological study, they can also lead to allergic sensitization and asthma development [136]. Parainfluenza viruses, coronaviruses, adenoviruses, and the newly discovered bocaviruses and metapneumoviruses are also involved, but they are less common [137,138]. Although it is unclear how viruses influence asthma onset, various studies have been carried out about the host response to respiratory viruses and how viruses can induce the host response or how subsequent allergen exposure and sensitization affect the host response.

### 7.1. Allergy Promoting Mechanism of Viruses

**Figure 3:**
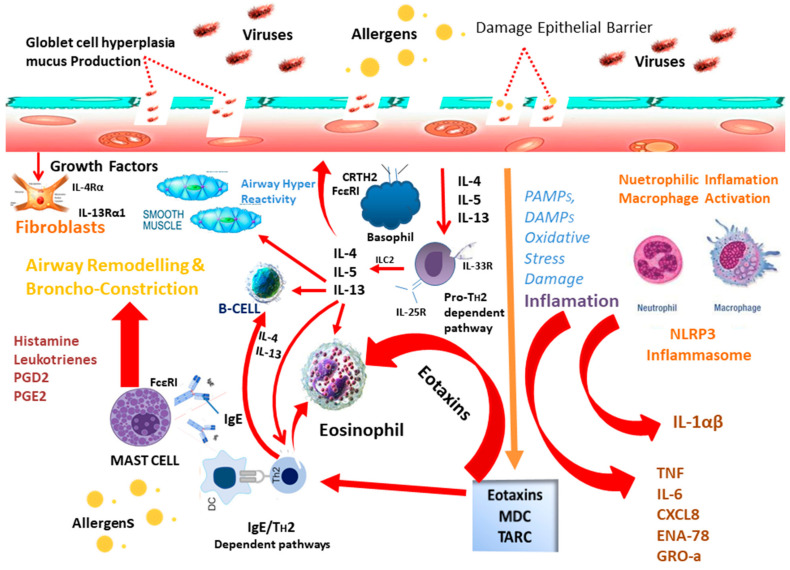
Overview of viral infection and synergistic or additive effects of allergen exposure causing IgE/T_H_2 and proT_H_2 inflammation. IL-25R: IL-25 receptor; GRO-a: melanoma growth stimulating activity-a; IL-13R: IL-13 receptor; IL-33R:IL-33 receptor; ENA-78: epithelial-derived neutrophil-activating protein 78; IL-4R: IL-4 receptor.

Although the mechanisms underlying the association of asthma and viral respiratory tract infection are not completely understood, recent reports indicate that epithelial cell viral infection could produce cytokines, such as IL-33 and IL-25, that interact with allergic inflammation, inducing both antigen-specific and innate T_H_2 cell–related pathways, and resulting in mucin production, increased T_H_2 related inflammation, enhanced IL-13, IL-4, and IL-5, and eosinophilia [139,140]. In patients with atopic asthma, the T_H_2 cytokines (IL-13, IL-5, and IL-4,) are well known as effector molecules [134]. The effects of viruses and allergens on immune and airway epithelial cells, as well as the elicitation of T_H_2 responses, are summarized in Figure 3.

Viral infection disrupts the epithelial barrier, resulting in the thymic stromal lymphopoietin and pro-T_H_2 cytokines IL-25 and IL-33. These cytokines act on T_H_2 cells, DCs, and ILC2s, causing the T_H_2 cytokines IL-13, IL-5, and IL-4 to be produced. These cytokines are important in asthma: IL-13 and IL-4 promote antibody class switching to IgE in B cells, IL-13 can also act on smooth airway muscle cells, causing bronchoconstriction and aiding in the remodeling of airways, and IL-5 stimulates the production of eosinophil. IL-4, IL-13, and virus actions on airway epithelial cells can elicit eotaxins, which attract eosinophils, as well as activation-regulated chemokine (TARC), the chemokines macrophage-derived chemokine (MDC), and thymus, which attract T_H_2 cells into the airway. IgE cross-linkage with allergens in mast cells releases leukotrienes, histamine, and the prostaglandins PGE2 and PGD2, which promotes bronchoconstriction. PGD2 activates ILC2s, T_H_2 cells, and basophils by binding to CRTH2, a chemoattractant receptor-homologous molecule expressed on T_H_2 cells. Oxidative stress can also be caused by viruses, and the formation of pathogen-associated molecular patterns (PAMP) and damage-associated molecular patterns (DAMP) can lead to pro-inflammatory cytokines, such as IL-6, TNF, and IL-1a/b. Propagative cytokines are generated. This usually results in macrophage activation and neutrophilic inflammation. Allergen-induced IL-1a can also stimulate the pro-T_H_2 response, resulting in ILC2 activation and IL-33 production [141].

### 7.2. Allergy and COVID-19

COVID-19, caused by SARS-CoV-2 [142,143], shares many symptoms with allergic diseases, such as coughing, olfactory, shortness of breath, nasal congestion, and taste dysfunction [144,145]. Some allergic disorders, such as chronic rhinosinusitis with nasal polyps (CRSwNP), allergic rhinitis, and asthma, can simulate COVID-19 symptoms: asthmatic patients experience cough and dyspnea, whereas allergic rhinitis and CRSwNP patients experience runny noses and headaches [146]. Multiple pathophysiological processes indicate that allergies may increase the risk of SARS-CoV-2 infection [141]. The respiratory virus initiates a local inflammatory cascade, resulting in cytokine production, which can aggravate asthma and allergy symptoms [139]. Besides, allergic patients have impaired innate interferon secretion, increasing their susceptibility to respiratory viral infections [141]. Besides, this pandemic began in the spring, when seasonal allergy sufferers are most likely to experience some of the same symptoms [147]. Chronic airways diseases and the COVID-19 pandemic are both associated with anxiety, which should be considered when interpreting subjective symptoms of both conditions. COVID-19 patients have been documented to have skin symptoms and signs of eczema and urticaria that are similar to acute urticaria or medication reactions, creating a diagnostic difficulty for allergists and dermatologists. For this reason, it is important to pay attention to COVID-19-specific symptoms, such as mainly fever, as well as excessive fatigue and a diminished sense of taste or smell, in order to make an accurate diagnosis [146,147].

Adults and children with allergies are more prone to have physical and mental health concerns during the COVID-19 pandemic. COVID-19 and allergens are independently associated with mental health problems [148,149,150]. Gonzalez-Diaz, et al. reported that patients with allergic diseases were more affected psychologically by the COVID-19 quarantine than those without allergies, as allergic individuals had a higher risk of depression symptoms [151]. Allergic patients were more likely to engage in various COVID-19 preventive measures, including maintaining a six feet social distance, avoiding crowded or public places, wearing a face mask, postponing or canceling activities, avoiding contact with high-risk people, and sanitizing or washing their hands [152]. A greater adherence to COVID-19 preventative activities showed the significant impact of the pandemic on the mental health of this group, because social isolation can cause hopelessness and depression [153]. Interactions between COVID-19 and allergy-related inflammatory psychiatric disorders, such as anxiety, post-traumatic stress disorder (PTSD), and depression, have been reported [154]. The stress of the COVID-19 pandemic, therefore, may increase the psychiatric reaction in those who have preexisting allergic conditions.

The COVID-19 epidemic has been a burden for allergy professionals. Since COVID-19 shares similar allergy disease symptoms, a pandemic may cause a problem in prioritizing allergic people, face-to-face assessment, and further concerns about the potential diagnostics of COVID-19. Face-to-face and hospital visits should be kept to a minimum for allergic disease patients, and more attention and promotion should be given to social distancing, hand disinfection, patient consultation adaptations, and sufficient PPE for health care employees. Teleconsultation for allergic patients during COVID-19 is very promising, and telemedicine platforms can provide a trustworthy service [155,156].

## 8. Bacterial Infections in Allergy

Bacteria play a dual role in allergies. They mainly concern protection, although certain species of bacteria stimulate allergic inflammation. Bacterial exposure has long been linked to allergy prevention. For example, mycobacteria are potent inducers of Th1 responses, notably IFN-release, which counteract type 2 inflammation and elicit regulatory T cell (Treg) responses, the primary anti-allergic immunological mechanism [87]. *Mycobacterium tuberculosis* infection and vaccination with other mycobacteria or *Bacillus Calmette-Guérin* reduce allergy prevalence in animals and humans [157,158,159]. Furthermore, there is a wealth of evidence suggesting bacterial compounds influence the innate immune system. TLR4 and other innate pattern recognition receptors play an important role in anti-allergenic effects [160].

In recent decades, the hygiene theory has been bolstered by the discovery of a considerable decline in infectious diseases associated with a sharp increase in the frequency of allergy: “The decline in the incidence of infectious diseases in industrialized countries throughout the past three decades is the major explanation for the increased prevalence of allergy diseases in those countries” [161]. As the role of commensal microbiota in immune regulation and inflammatory homeostasis became more apparent, this hypothesis was later modified. Early exposure to innocuous endogenous and exogenous microorganisms reduces allergy risk. In general, the alterations in the microbiome might affect allergy manifestations, both in terms of their diversity and abundance [162,163,164]. Due to this observation, the ability of specific commensal gut microflora species (probiotic strains) to promote immunological tolerance, notably lactic acid bacteria, including Bifidobacteria or Lactobacillus, is currently being examined. Several reports exist that detail the significance of these strains in allergy disorders prevention [165,166,167].

On the other hand, epidemiological evidence suggests that infection or colonization with specific bacterial species might cause or worsen allergies [163,168]. Bacteria, for example, can aggravate asthma symptoms on their own or in combination with viruses, such as the respiratory syncytial virus or human rhinovirus [169,170]. Studies in the 1970s and 1980s showed that bacterial colonization was linked with allergy disorders. Atypical bacteria, such as *Mycoplasma pneumonia*, *Chlamydia pneumoniae*, and *Chlamydia trachomatis*, have been associated with asthma exacerbations, lung remodeling, and an increased incidence of wheezing episodes. These pathogens have also been found in nasal washes, sera, and bronchoalveolar lavage fluid (BAL) from asthmatic patients [171,172,173,174]. Infection or colonization with *Streptococcus pneumoniae, Staphylococcus aureus, Moraxella catharralis,* and *Haemophilus influenzae,* among the common human respiratory tract bacterial inhabitants, has been linked to recurrent wheezing in children, obstructive pulmonary disease, and the onset and exacerbation of asthma, [169,170,175,176]. Furthermore, the asymptomatic *M. catarrhalis* or *S. pneumoniae* colonization of newborns is linked to asthma development and a recurrent wheeze later in life [175].

### Allergy Promoting Mechanism of Bacteria

Bacteria have been found to exhibit a variety of pro-allergenic activities (non-antigen-specific and antigen-specific). Airway epithelial cells can be infected by bacteria, causing cell death, inflammation, and the breakdown of the epithelial barrier. Pore-forming toxins, such as bacterial proteases and *S. aureus* toxins (Hla), also play a role in epithelial barrier breakdown. Microbial invasion is facilitated by an increased epithelial permeability, which exposes the immune system to allergens and environmental pollutants [177].

Some bacteria can cause histamine release from mast cells and human basophil leukocytes via independent or IgE-dependent mechanisms [178,179]. Several studies have shown that bacteria can cause both naive T cells to differentiate into T_H_2 or T_H_17 cells and the release of T_H_2 cytokines [178,180,181].

The induction of cytokines type 2 is likely to result in a switching of the Ig class to IgE. IgE antibodies directed against *C. pneumoniae, C. trachomatis, H. influenzae, S. aureus, M. pneumoniae, M. catharralis,* or *S. pneumonia* have been described [171,175,181,182]. Despite the fact that antibacterial IgE can be measured, there is evidence of allergy protection from exposure to *S. pneumonia* or *H. influenzae*. Specific IgE antibodies were found to be inversely linked to asthma risk in teens in diverse proteins of these microbial species. Furthermore, they emphasize the significance of the mechanistic and epidemiological validation of allergen prediction [183].

House dust mites (HDM) have recently been shown to be antigen carriers for bacteria colonizing the respiratory tract, skin, or gut, such as *E. coli* or *S. aureus*. Hence, HDM could cause or aid bacterial antigen sensitization. This may help to explain why the IgE response to bacterial antigens is so common in skin and respiratory allergy symptoms [184].

**Figure 4:**
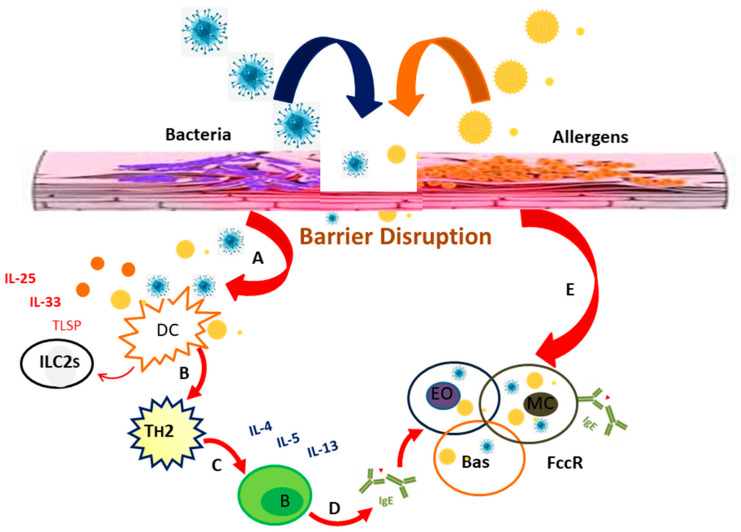
Allergy inducing mechanisms of bacteria. * Eo, eosinophil; FCεR, high affinity IgE receptor; B, B cell; Bas, basophil; TSLP, thymic stromal lymphopoietin; MC, mast cell; ILC2, innate lymphoid cell type 2; DC, dendritic cell; Th, T helper cell.

Hence, bacteria exert control over general allergy-inducing pathways and may become targets for type 2 immune responses defined by IgE antibodies and specific T_H_2 cells [183]. Figure 4 depicts an overview of bacteria’s allergy-promoting processes.

Bacteria have described a number of pro allergenic pathways. Bacterial toxins and proteases break down the epithelial barrier, allowing for the entry of conventional allergens and microbial invasion. This causes the release of potent immune mediators (TSLP, IL-33, and IL-25) and local inflammation (a). This pathway helps to recruit and differentiate naive T-cells into effector T-cells (T_H_17 and T_H_2), which leads to the release of pro-allergenic Th2 cytokines. Type 2 cytokine secretion is also elicited by tissue-resident ILC2s (b). B cells differentiate into IgE-secreting plasma cells after undergoing an Ig class switch (c). IgE promotes the activation and recruitment of basophils, mast cells, and eosinophils (d). Bacterial components can potentially cause the IgE-independent degranulation of these effector cells, increasing allergic inflammation (e) [183].

## 9. Treatments

Advances in allergy research have made a significant impact on the treatment of moderate to severe allergic disorders. Numerous treatments for different symptoms of allergic disorders, as well as several drugs that effectively control and treat atopic conditions, are available. Epinephrine shots for anaphylaxis are available and can be carried with the patient, while anti-inflammatory and antihistamine medicines are commonly administered to relieve symptoms in others [185,186].

The treatment of allergic diseases in children follows a similar pattern to that of adults. Treatment options include allergen avoidance through environmental control, pharmacotherapy, and immunotherapy. The main goal of treatment is to control symptoms without affecting the child’s functioning. The second, but equally important, goal is to prevent the development of the sequelae of allergic diseases. Currently, the best approach for a child at a high risk of developing allergies is to implement dietary and environmental control measures early in order to reduce sensitization, and to acknowledge and treat the signs and symptoms of allergic disease as they emerge [187,188,189].

Various methods of diagnosis have been created based on allergen sources, and therapy strategies based on diagnostic methods have been developed to address allergic reactions concerns. Some of the treatments utilized for allergic diseases are the following.

### 9.1. Allergen Avoidance

The primary focus of allergy treatment should always be the strict avoidance of specific allergens that cause allergic disease. The greatest and best guideline for reducing allergy reactions in sensitive people is to avoid allergen exposure. Food allergies and some stinging insect allergies are treated primarily through avoidance, which can be quite helpful if patients are well trained about preventive measures. However, it is impossible to avoid certain allergens that travel through the air and are easily inhaled without control or notice. Avoidance is impossible in these circumstances, and additional therapeutic procedures are necessary to overcome difficulties [16,190].

### 9.2. Pharmacotherapy

Pharmacotherapy can relieve allergen-induced symptoms when allergen prevention and tracking are impossible and allergy exposure is inevitable. Many drugs are developed that are antagonistic to and block the actions of allergic mediators. Anti-leukotrienes and antihistamines are two common drug targets that prevent the onset of allergic symptoms and inhibit the action of inflammatory mediators [185,186]. The FDA has approved adrenaline (epinephrine), antihistamines, glucocorticosteroids, and theophylline, which primarily act as anti-inflammatory molecules. Decongestants, mast cell stabilizers, and eosinophil chemotoxins, along with anti-leukotrienes, such as zafirlukast (Accolate) or montelukast (Singulair), are commonly used as drugs to monitor and prevent chronic and acute allergic diseases.

### 9.3. Immunotherapy

Allergen-specific immunotherapy entails administering an increasing dose of allergens to a patient over time to ensure immunological and clinical tolerance. Allergen injection immunotherapy induces T cell tolerance through a variety of methods, including alteration in secreted cytokines, decreased allergen-induced proliferation, stimulation of apoptosis, and T regulatory cells production. This results in the reduction of inflammatory mediators and cells in the affected tissues, production of blocking antibodies, and suppression of IgE [191]. This sort of immune therapy has been demonstrated to be effective in studies, and long-term use has indicated that immunotherapy can help to avoid the development of atopy. The intravenous administration of monoclonal anti-IgE antibodies is the second type of immunotherapy. These attach to both B-cell-associated and free IgE, signaling and killing them [192]. Sublingual immunotherapy is a third type of therapy that is given orally and is based on oral immune tolerance to non-pathogens, such as resident bacteria and foods. Allergy shot therapy may become the most effective allergy treatment method in the future. Close supervision and a long-term commitment are required in this therapy for successful individual treatment [193].

### 9.4. Ineffective and Unproven Treatments

An enzyme potentiated desensitization (EPD) experimental treatment has been tested in some recent investigations, but no encouraging outcomes have been found. The same method is currently used in many hypoallergenic food preparations. The treatment approach, however, was not convincing, and was not acknowledged as effective. EPD uses allergen dilutions with beta-glucuronidase enzymes to polarize T-regulatory lymphocytes and to change the allergen nature, which down-regulates IgE induction, favors desensitization, and prevents allergic reactions [194].

## 10. Role of Bioinformatics in Allergic Diseases Management

Allergy research has progressed quickly in recent years [195]. Recent advances in proteomics, analytical methods, and genomics have resulted in massive amounts of allergen-related data. The pathophysiology of many allergy conditions based on epidemiologic, experimental, and clinical information for allergic reactions can be related to this data. A continuous data increase requires effective archival, data management, and data analysis. In the modern era, bioinformatics applications are used to predict allergens and their allergenicity. Bioinformatics complements wet-lab research by providing tools for managing this avalanche of data. Despite the fact that a large amount of biological data is difficult to manage, specific tools and databases are available to handle data. Several tools, databases, and servers contain a wide range of information about allergens and other potential side effects. The goal of allergy-related databases is to make data retrieval, collection, and analysis easier. Furthermore, bioinformatics techniques can be used to organize allergens and to identify areas that may account for common IgE binding patterns and cross-reactivity [196]. These findings can be used to help allergy sufferers choose the best treatment options. Hence, the discipline of allergy bioinformatics has emerged, which includes allergen-specific resources/databases, as well as computational tools/methods [197]. Many research papers on allergen bioinformatics and immunoinformatics have been published by various groups of researchers [198,199]. For example, Zhang et al. identified key genes and Le Chen et al. identified hub genes in a murine model in allergic rhinitis by bioinformatics analysis [200,201]. Deocaris et al. used bioinformatics analysis in the detection of nascent allergens in GMO and conventional rice [202]. L’Hocine et.al identified allergens from Canadian mustard varieties of Brassica juncea and Sinapis alba [203]. Chenbei et.al performed a bioinformatics analysis of the dataset to identify pathways and potential different expressed genes (DEGs) related to childhood allergic asthma [204].

Allergen bioinformatics deals with tools/algorithms for allergenicity/allergen prediction, allergenic cross-reactivity prediction, allergen databases, and allergen epitope prediction [197].

### 10.1. Allergen Cross-Reactivity Prediction

Cross-reactivity has a major role in clinical and immunological allergic reactions. The prediction of cross-reactivity in allergy was therefore considered to be significant [205]. In the majority of cases, the prediction of allergenicity is associated with the allergens cross-reactivity prediction. This is due to the fact that the antigenic determinants that cause allergen cross-reactivity are also responsible for allergenicity [206]. Hence, many of the algorithms/tools designed to predict allergen/allergenicity can also predict cross-reactivity. The criteria set by FAO/WHO experts aid in the allergen cross-reactivity identification [207]. Stadler and Stadler [208] proposed a sequence-based approach and claimed that a motif-based strategy outperforms the WHO/FAO guidelines for cross-reactivity calculations. AllerTool [209] is a cross-reactivity webserver based on the WHO/FAO guidelines and amino acid sequence. It also depicts published and projected allergen cross-reactivity patterns graphically. A sequence-based technique for determining the allergen cross-reactivity is included in SDAP [210], a specialized allergen database. AllerHunter [211] is a web server based on SVM that efficiently analyzes allergen cross-reactivity in proteins. A recently developed algorithm for an allergenicity prediction based on a fuzzy inference system can also predict allergen cross-reactivity [212].

### 10.2. Allergen Databases

Significant technological advances in the fields of proteomics and genomics, as well as considerable improvements in analytical methods, have occurred in recent years. As a result, significant progress has been made in allergy research. Hence, the number of identified protein allergens has been steadily increasing in recent years [213,214]. As a result of the constant accumulation of allergen-related clinical and molecular data, efficient data storage and management has become critical. Allergy databases are therefore very essential resources for fundamental allergy research because they are used to archive available knowledge about allergens [215]. Table 2 provides a summary of allergen-specific databases.

### 10.3. Allergen/Allergenicity Computational Prediction

Allergens are primarily proteins found in pollens, food, and other biological entities in the environment. As a result of the health risks associated with allergic reactions to these proteins, it has become necessary to evaluate their potential allergenicity. Food processing and genetic engineering methods have been frequently used in recent years to modify the existing or new proteins. Analyzing the allergenicity of such products/proteins and biopharmaceuticals is critical to avoid the allergenic molecule transfer. The most common method for assessing allergenicity is computational prediction or evaluation, and a range of bioinformatics methods/tools have been effectively used for this purpose [223]. Most of these strategies are based on amino acid sequences and their different properties, with only a few approaches based on structural information [224]. The list of computational servers/tools available for the allergenicity/allergen prediction is shown in Table 3.

### 10.4. Allergens Epitopes Computational Prediction

Epitopes are distinguishing amino acid residuals on antigens and are significant predictors of immune responses. The identification of epitopes is considered to be a key step in the creation of effective multi subunit vaccines, as well as efficient therapeutic and allergy diagnostic procedures. IgE binding epitopes, also known as B cell epitopes, are proteins that recognize IgE binding sites in allergens. They play a significant role in the interaction between the allergen and IgE antibody. IgE-binding epitopes have distinct characteristics that distinguish the antibody epitope from other epitopes. Complex allergens and antibodies are widely used in allergen immunotherapy and aid in the understanding of allergy phenomena. There are a large number of epitopes in databases that can be used as a template for novel epitope predictions [229].

The antigen contains T cell epitopes; it is also known as the antigenic determinant that interacts with the T cell through T cell receptors. Allergen T cell epitopes have been discovered to play an important role in allergic reactions. As a result, the identification of allergic disorders would be cured by developing new epitope-based immunotherapy, which would allow for the production of effective vaccines [229].

Although experimental methods have proven to be effective in discovering epitopes, their utility is restricted due to their high cost and time requirements, as well as their incapacity to deal with large-scale epitope elucidation. Computational approaches are therefore deemed highly useful because they are cost effective and time efficient. A wide range of highly effective algorithms and methods for the prediction of epitopes have been developed throughout the years. Both T cell and B cell epitopes, including discontinuous (conformational) and sequential (linear) epitopes, are predicted using these approaches [230,231]. Table 4 lists some of the most popular tools and servers for B cell and T cell epitopes prediction.

## 11. Conclusions and Future Perspectives

Allergies are a severe problem that affects millions of individuals throughout the world. It may be difficult to avoid offending allergen exposure if the causative allergen is rare or unknown. Allergic patients can, however, minimize symptoms by avoiding allergen exposure. Currently, available diagnosis and treatment methods aim to alleviate symptoms; however, medication would not provide long-term relief from allergic disorders. Researchers are conducting new studies and investigations to find solutions for allergy treatment. Advances in analytical, proteomic, and genomic approaches have resulted in a massive amount of data concerning allergens and allergies. In allergen bioinformatics, analyzing and archiving these data poses a significant challenge. The tools and resources of bioinformatics play a crucial role in overcoming this challenge. With the ever-increasing volume of data, it is critical to focus on the development of resource/databases that will integrate and provide quick access to information from literature and other sources. An analysis of such data can be used to have a clear understanding of allergic reactions. Allergen structural properties influence allergenicity significantly; this knowledge is used to develop effective methods for predicting allergen cross-reactivity and allergenicity/allergen. Recent epitope prediction advancements have focused on antibody-specific epitope prediction methods. The use of these techniques for IgE-binding epitopes predictions will be critical in the development of better and more efficient strategies for allergic disease treatment and diagnosis. Allergen immunotherapy (AIT), a treatment approach based on allergens, has been regarded as a prototype of personalized medicine or precision medicine. Bioinformatics could play a significant role in the development of breakthrough AIT methodologies and in the advancement of allergen bioinformatics. This will certainly contribute to a better knowledge of allergy diseases and will have a beneficial impact on future research in the field.

## Figures and Tables

**Table 1:** Symptoms, causes, and prevalence of different allergy types.

Type of Allergy	Symptoms	Prevalence	Affected Organ	Causes	Reference
Allergic rhinatisis	Sneezing, itchy, watery, and red eyes, stuffy or runny nose, swelling around the eyes.	Affects 10–30% of the population worldwide	Nose	Genetic and environmental factors	[19]
Asthma	Wheezing, coughing, shortness of breath, and chest tightness	Affects 3 to 9% of the population worldwide	Airways of lungs	Genetic and environmental factors	[20]
Food allergy	Itchiness, vomiting, swelling of the tongue, hives, diarrhea, low blood pressure, trouble breathing	Affects 8% of the population worldwide	Skin, respiratory system, gastrointestinal tract	Immune response to food	[21]
Skin allergy	Rash, itching, swelling, redness, cracked skin, flaking or scaling of skin, raised bumps	Worldwide, lifetime prevalence of above 20%	Skin	Latex, food, drugs, water, sunlight, nickel, chemicals, soap, poison oak or poison ivy	[22]
Drug allergy	Itching, rash, fever, facial swelling, hives, shortness of breath, cardiac symptoms	Affects 10% of the population worldwide	Nose, lungs, throat, ear, lining of the stomach, and skin	Reactions to medications	[23]
Insect allergy	Itching, pain, and swelling and appearance of redness at the sting/bite or surrounding affected areas	Many allergic severe cases have been documented with insect bites worldwide; however, there has been no systemic report.	Skin, eyes, throat, tongue	Insects bite or sting	[24]
Anaphylaxis	Itchy rash, numbness, throat swelling, lightheadedness, shortness of breath	Affects 0.05–2% of the population worldwide	Skin, nose, throat, lungs, gastrointestinal tract	Foods, insects bites, medications	[25]

**Table 2:** Details of allergen-specific databases.

Database	URL	Maintained by	Type of Data Archived	Last Update	Number of Allergens/Haptens/Epitopes	Reference
IUIS Allergen	http://www.allergen.org (Accessed on: 19 August 2021)	World Health Organization (WHO) and International Union of Immunological Societies (IUIS)	Allergenicity, structure, sequence (isoforms/isoallergens)	Updated continuously	853	[216]
Structural Database of Allergenic Proteins (SDAP)	https://fermi.utmb.edu (Accessed on: 19 August 2021)	Sealy Centre for Structural Biology, University of Texas, USA	IgE epitopes, structure, sequence, structural models	2013	1526	[210]
AllerBase	www.bioinfo.net.in/AllerBase/Home.html (Accessed on: 19 August 2021)	Bioinformatics Centre, Savitribai Phule PuneUniversity, India	Structure and sequence (cross-links), IgE epitopes, IgE cross-reactivity, experimental evidences of allergenicity, IgE antibody	Updated continuously	2311	[217]
AllFam	http://www.meduniwien.ac.at/allfam (Accessed on: 19 August 2021)	Department of Pathophysiology and Allergy Research, Medical University of Vienna, Austria	Cross-link to Pfam database, allergen family data	2011	936	[218]
Allergen Online	http://www.allergenonline.Org (Accessed on: 20 August 2021)	Food Allergy Research and Resource Program (FARRP) at the University of Nebraska-Lincoln	Allergenicity, sequence	2016	1956	[219]
Allergen Database For Food Safety (ADFS)	http://allergen.nihs.go.jp/ADFS/ (Accessed on: 20 August 2021)	National Institute of Health, Japan	IgE epitopes, sequence, structure, small molecule allergens	2016	2028	[220]
AllergenPro	http://nabic.rda.go.kr/allergen (Accessed on: 20 August 2021)	The National Agricultural Biotechnology Information Center (NABIC), Korea	IgE epitopes, sequence	2015	2434	[221]
Allergome	http://www.allergome.org (Accessed on: 20 August 2021)	Centre for Clinical and Experimental Allergology, Italy	Sequence (isoforms/isoallergens), clinical, cross-reactivity, structure, epidemiologically	Updated continuously	3075	[222]

**Table 3:** Tools/servers for allergen/allergenicity prediction.

Server	URL	Method	Efficiency	Reference
AlgPred	http://www.imtech.res.in/raghava/algpred/ (Accessed on: 21 August 2021)	SVM and allergen sequence features, epitopes, sequence motifs	Accuracy: 85% Specificity: 88.1%	[4]
AllerHunter	http://tiger.dbs.nus.edu.sg/AllerHunter/ (Accessed on: 21 August 2021)	SVM and iterative pairwise sequence similarity	Accuracy: 95.3% Specificity: 96.41%	[211]
PREAL	http://gmobl.sjtu.edu.cn/PREAL/index.php (Accessed on: 21 August 2021)	Physicochemical and biochemical descriptors, sequence features, subcellular locations, SVM, and mRMR	Accuracy: 93.42%	[225]
AllergenFP	http://ddg-pharmfac.net/AllergenFP/ (Accessed on: 21 August 2021)	Descriptor-based fingerprints of residues, auto and cross-covariance	Accuracy: 88%	[226]
AllerTOP	http://www.ddg-pharmfac.net/AllerTOP (Accessed on: 22 August 2021)	Machine learning, sequence based descriptors, cross and auto -covariance,	Accuracy: 85.3% Specificity: 88.1%	[227]
AllerCatPro	https://allercatpro.bii.a-star.edu.sg/ (Accessed on: 22 August 2021)	Sequence similarity, structure similarity	Accuracy: 84% Specificity: 67%	[228]

**Table 4:** Tools/servers for B cell and T cell epitopes prediction.

Type	Server	URL/Website	Method	Reference
Linear B cell Epitope	ABCPred	(http://www.imtech.res.in/raghava/abcpred/) (Accessed on: 25 August 2021)	ANN	[232]
	BepiPred	(http://www.cbs.dtu.dk/services/BepiPred/) (Accessed on: 25 August 2021)	HMM	[233]
	LBtope	(http://www.imtech.res.in/raghava/lbtope/) (Accessed on: 25 August 2021)	SVM	[234]
	BCPreds	(http://crdd.osdd.net/raghava/bcepred/) (Accessed on: 25 August 2021)	SVM	[235]
	BEST	(http://biomine.ece.ualberta.ca/BEST/) (Accessed on: 25 August 2021)	SVM	[236]
	SVMTriP	(http://sysbio.unl.edu/SVMTriP/) (Accessed on: 25 August 2021)	SVM	[237]
Conformational/Discontinuous B cell Epitope	DiscoTope 2.0	(http://www.cbs.dtu.dk/services/DiscoTope/) (Accessed on: 27 August 2021)	Structure-based method	[238]
	B-Pred	(http://immuno.bio.uniroma2.it/bpred) (Accessed on: 27 August 2021)	SVM	[239]
	ElliPro	(http://tools.immuneepitope.org/tools/ElliPro) (Accessed on: 27 August 2021)	Thornton’s method	[240]
	CBTOPE	(http://crdd.osdd.net/raghava/cbtope/) (Accessed on: 27 August 2021)	SVM	[241]
	EpiPred	(http://opig.stats.ox.ac.uk/webapps/newsabdab/sabpred/epipred/) (Accessed on: 27 August 2021)	Structure-based method	[242]
T cell Epitope	EpiTOP	(http://www.pharmfac.net/EpiTOP/) (Accessed on: 29 August 2021)	QSAR	[243]
	CTLPred	(http://www.imtech.res.in/raghava/ctlpred/index.html) (Accessed on: 29 August 2021)	ANN, SVM	[244]
	PREDIVAC	(http://predivac.biosci.uq.edu.au/) (Accessed on: 29 August 2021)	MM	[245]
	MHCPred	(http://www.ddgpharmfac.net/mhcpred/MHCPred/) (Accessed on: 29 August 2021)	QSAR	[246]
	NetMHCIIpan-3.0	(http://www.cbs.dtu.dk/services/NetMHCIIpan-3.0/) (Accessed on: 29 August 2021)	ANN	[247]

## Data Availability

The data presented in this study are available within the article.
